# Clinical spectrum of extreme insulin resistance syndromes treated with rhIGF-1: A single-center experience

**DOI:** 10.1210/clinem/dgag031

**Published:** 2026-01-28

**Authors:** Nicola Improda, Harshini Katugampola, Manuela Cerbone, Pratik Shah, Saji Alexander, Abigail Atterbury, Smail Hadj-Rabia, Catherine J Peters, Robert K Semple, Mehul Tulsidas Dattani

**Affiliations:** Neuroscience Department, Neuroendocrine and Obesity Centre, Santobono-Pausilipon Children’s Hospital, Naples 80139, Italy; Department of Medical Translational Sciences, Federico II University of Naples, Naples 80131, Italy; London Centre for Paediatric Endocrinology and Diabetes at Great Ormond Street Children’s Hospital, London WC1N 1EH, UK; London Centre for Paediatric Endocrinology and Diabetes at Great Ormond Street Children’s Hospital, London WC1N 1EH, UK; Section of Molecular Basis of Rare Disease, Genetics and Genomic Medicine Research and Teaching Department, University College London Great Ormond Street Hospital Institute of Child Health, London WC1N 1EH, UK; Paediatric Endocrinology Department, The Royal London Children’s Hospital and Queen Mary University of London, Barts Health NHS Trust, London E1 1BB, UK; Department of Paediatric Endocrinology and Diabetes, Chelsea and Westminster NHS Foundation Trust, London SW10 9NH, UK; London Centre for Paediatric Endocrinology and Diabetes at Great Ormond Street Children’s Hospital, London WC1N 1EH, UK; Department of Dermatology and Reference Center for Rare Skin Diseases, Hôpital Necker-Enfants Malades, AP-HP Centre Université Paris Cité, Paris 75015, France; INSERM U1163, Institut Imagine, Hôpital Universitaire Necker-Enfants Malades, Paris 75015, France; London Centre for Paediatric Endocrinology and Diabetes at Great Ormond Street Children’s Hospital, London WC1N 1EH, UK; Centre for Cardiovascular Science, The University of Edinburgh, Edinburgh EH16 4TJ, UK; MRC Human Genetics Unit, Institute of Genetics and Cancer, The University of Edinburgh, Edinburgh EH4 2XU, UK; London Centre for Paediatric Endocrinology and Diabetes at Great Ormond Street Children’s Hospital, London WC1N 1EH, UK; Section of Molecular Basis of Rare Disease, Genetics and Genomic Medicine Research and Teaching Department, University College London Great Ormond Street Hospital Institute of Child Health, London WC1N 1EH, UK

**Keywords:** Donohue syndrome, Rabson-Mendenhall syndrome, insulin resistance, recombinant human insulin-like growth factor-1, hyperinsulinemic hyperglycemia, diabetes mellitus

## Abstract

**Context:**

Donohue syndrome (DS) and Rabson-Mendenhall syndrome (RMS) are extreme forms of insulin resistance (IR) caused by biallelic mutations in the insulin receptor gene. Recombinant human insulin-like growth factor-1 (rhIGF-1) treatment is often used but its long-term benefits and risks are still poorly delineated.

**Objective:**

We describe rhIGF-1 treatment outcomes of a cohort of patients with DS and RMS, and compare them to previously published patients.

**Methods:**

A single-center retrospective observational study with case note review was conducted. A literature search was performed for previously published DS and RMS patients treated with rhIGF-1.

**Results:**

rhIGF-1 outcomes beyond 4 months have been reported for only 11 patients with DS and RMS to date. We provide outcome data for 3 more males, and report the long-term clinical course of a patient already described. Metabolic benefits of rhIGF-1 included improved glycemic control and fasting tolerance in early years of life, and some growth enhancement. Two patients exhibited poor response or intolerance to rhIGF-1 and died in infancy. The 2 longest-lived patients had progressive decompensation to diabetes mellitus, in 1 case despite long-term uninterrupted rhIGF-1 therapy. We also describe new features associated with severe insulin receptoropathies, such as cataract, liver hemangioma, and impaired hepatic protein synthesis.

**Conclusion:**

The phenotypes of DS and RMS are heterogeneous. Current treatment options remain unsatisfactory as high-dose rhIGF-1 exerts only limited beneficial effects and does not prevent decompensation to diabetes mellitus. More research is needed to identify alternative treatment strategies for extreme forms of IR.

Donohue syndrome (DS) and Rabson–Mendenhall syndrome (RMS) are recessive conditions that form the extreme end of a continuum of insulin resistance (IR) due to genetic insulin receptor (INSR) defects. They are not precisely defined, but DS is usually associated with death in the first years of life, whereas RMS is associated with longer survival (1). Their incidence is estimated at 1:1 000 000 live births (2). DS and RMS are clinically characterized by prenatal and postnatal growth restriction, facial dysmorphism, paucity of adipose and muscular tissue, soft tissue overgrowth, acanthosis nigricans, and hirsutism. Biochemical features include fasting hypoglycemia and postprandial hyperglycemia, with resistance to ketoacidosis in the first years of life that progressively yields to ketoacidosis-prone diabetes mellitus (1). Renal tubular dysfunction with nephrocalcinosis is almost invariably present (3, 4), and case reports attest to numerous other biochemical and clinical comorbidities in some cases, but the rarity of the conditions means that the full spectrum of these is not well defined.

Recombinant human insulin-like growth factor-1 (rhIGF-1) has been used off-label over the last 30 years in an attempt to mitigate the metabolic disorder associated with the condition. rhIGF-1 binds to the IGF-1 receptor (IGFR), INSR, and INSR/IGFR hybrid receptors (5, 6), activating post–receptor signaling pathways that are partially shared with insulin (1). This treatment exerts proven short-term metabolic benefits in patients with extreme forms of IR (7). On the other hand, current evidence regarding the long-term benefits is sparse and difficult to interpret due to different types of potential biases, lack of controlled studies, and functional studies of only a small number of *INSR* pathogenic variants. The optimal treatment regimen remains to be established, as does the magnitude of any likely potential benefit (1, 5). The results of a recent systematic review (8) assessing the outcomes of rhIGF-1 or IGF-1/IGFBP-3 combined treatment beyond 28 days in 7 biallelic carriers of pathogenic *INSR* variants showed an improvement in glycated hemoglobin A_1c_ (HbA_1c_) only, in the region of 1.5%. On the other hand, 2 hypoglycemic episodes and 1 case of soft tissue overgrowth were reported.

We report the clinical features and treatment outcomes of a single-center cohort of patients with DS/RMS, all of whom were treated with bolus rhIGF-1 for up to 16 years. We expand the knowledge of multisystem features of these rare syndromes, and place treatment outcomes in the context of long-term outcomes in all previously reported cases.

## Materials and methods

We conducted a retrospective search for all patients referred to Great Ormond Street Hospital for DS and RMS treated with rhIGF-1 over the last 20 years. Data related to auxology, organ damage, glycemic, lipid, hormonal, and coagulation profile were curated longitudinally from the first visit. INSR amino acid changes are numbered according to the proreceptor sequence of the B isoform of the insulin receptor (UniProt P06213-2), with mature receptor numbering in parentheses. Informed consent was obtained from all individuals and/or their guardians before participation. To compare treatment outcomes between our patients and previous reports, we performed a literature search in the PubMed database for other cases of DS or RMS treated with long-term rhIGF-1. Key words used were “mecasermin,” “rhIGF-1,” “severe insulin resistance,” “Donohue syndrome and IGF-1,” “Rabson-Mendenhall syndrome and IGF-1.”

## Results

Four patients (1 with RMS and 3 with DS) were treated with rhIGF-1 during the study period. The clinical features of these patients are summarized in Table 1. In addition, we found 12 reports in the literature of patients with syndromes of extreme IR (of whom 3 were defined as having RMS) treated with rhIGF-1 for at least 4 months. Ten out of 12 patients were genetically proven to have biallelic *INSR* mutations, while biallelic mutations were assumed in 2 further patients, 1 in whom only a monoallelic mutation was proven, and another with no proven mutations. As shown in Table 2, main treatment outcomes were reported only for 11 out of 12 patients.

**Table 1:** Clinical characteristics and treatment outcomes of the 4 patients with Donohue syndrome

Patient	1	2	3	4
*INSR* mutation	p.Arg1119Gln homozygote	p.Ile146Met/p.Arg1066X	p.Gly111Glu homozygote	p.Trp642Arg homozygote
Current age, y	NA	17	NA	NA
Comorbidities	Nephrocalcinosis, bilateral cataracts, ventricular hypertrophy, recurrent sepsis	Nephrocalcinosis, psoriasiform dermatitis with recurrent skin infections, glue ear, transient cholestatic jaundice, ventricular hypertrophy, reduced coagulation factor IX	Nephrocalcinosis, proximal renal tubulopathy, liver failure, coagulation factor and albumin deficit, ventricular hypertrophy, rectal prolapse, left inguinal hernia, secondary hypothyroidism	Nephrocalcinosis, hypoplastic anemia, recurrent sepsis, ventricular hypertrophy, patent ductus arteriosus, coagulation factor and albumin deficit, liver hemangiomas, secondary hypothyroidism
Age of death, y/cause	14.68/unknown (recurrent sepsis and ketoacidosis)	NA	0.9/liver failure; respiratory infection	2.3/wound breakdown and airway complications after abdominal surgery
Age at rhIGF-1 start, y	1.9; restart at 13.4	0.75 (IGF-1 + IGFBP3 until 8 y, then rhIGF-1)	0.1	0.1
Therapy duration, y	3.3 (total among 2 trials)	18	0.3	2
Starting rhIGF-1 dose, mg/kg/d Frequency	0.04; OD	0.05; BD	0.1; BD	0.08; BD
Maximum rhIGF-1 dose, mg/kg/d Frequency	0.64; BD	0.3; TDS	0.4; BD	0.27; TDS

	Pre–rhIGF-1/latest	Pre–rhIGF-1/latest	Pre–rhIGF-1/latest	Pre–rhIGF-1/latest
Height SDS	−7.1/−7.1	−1.96/−2.33	−2.1/−5	−1.25/−8.28
Weight SDS	−5.48/−4.2	−2.36/−3.45	−5/−4.95	−5.45/−6.13
Feeding regimen	Enteral (boluses)/enteral (boluses)	Enteral (continuous)/enteral (boluses)	Parenteral/parenteral	Enteral (boluses)/enteral (continuous)
Fasting tolerance, h	NA/ ≥ 12	2/ ≥ 12	1/2	2/2
HbA_1c_, %	18.7/11.5	4.9/9	NA, fetal Hb	5.9/7.5
Mean BG at CGMS, mmol/L	15.2/24.3	4.44/20	NA	6.15/11

Abbreviations: BD, 2 daily doses; BG, blood glucose; CGMS, continuous glucose monitoring system; Hb, hemoglobin; HbA_1c_, glycated hemoglobin A_1c_; NA, not available; OD, once daily; rhIGF-1, recombinant human insulin-like growth factor-1; TDS, 3 daily doses.

**Table 2:** Summary of the main studies reporting long-term (>4 months) treatment with mecasermin in Donohue and Rabson-Mendenhall syndromes

Study	No. of patients/original diagnostic label*^a^*/sex	Genetic background	Age at treatment start, y	Treatment duration, mo	Maximum rhIGF-1 dose, mg/kg/d	IGF-1 regimen (OD/BD/TDS/QDS/CSI)	Main treatment outcomes	Adverse events possibly related to drug	Duration of survival, y
Grasso et al, 2013 (4)	1/RMS/M	Biallelic	0.4	20	0.6	NA	NA	Nocturnal hypoglycemia (during pulmonary infection?)	>3
Kuzuya et al, 1993 (7)	1/DS/F 1/DS/F	Monoallelic Unknown	0.6 7	15 16	0.4 0.1	BD BD	Increased IGF-1 Reduced plasma insulin No changes in HbA_1c_ and fructosamine concentrations Improvement of acanthosis nigricans and hirsutism Increased subcutaneous fat mass and skin elasticity Improved linear growth	None	NA
Longo et al, 1994 (18) Longo et al 1999 (17)	1/RMS/M	Biallelic	2	16	0.1	OD	Increased IGF-1 Reduced plasma insulin No increase in fasting and postprandial glucose or IGFBP-3 No increase in linear growth or weight	Moderate hypertension Kidney enlargement	7
Takahashi et al 1997 (6) Early course of patient 1 in our study	1/DS/M	Biallelic	1	6	1	NA	Reduced glucose, insulin and HbA_1c_ Improvement of weight and neuromotor development No changes in acanthosis nigricans	None	15 (see patient 1 in present study)
Nakae et al, 1998 (5) Kitamei et al, 2005 (14) Jo et al, 2013 (10)	1/DS/F	Biallelic	0.5	216	1.6	TDS/CSI	Increased IGF-1 Slight increase in IGFBP-3 Decreased IGFBP-1 Decreased blood glucose and HbA_1c_ Improved linear growth (dose dependent)	Tonsillar hypertrophy Enlargement of kidney, spleen, ovary, extraocular muscles, and lacrimal glands Severe retinal neovascularization Mastitis Endometrial cancer	>24
Weber et al, 2014 (15)	1/DS/F	Biallelic	1.7	16	1.2	QDS/CSI	Increased IGF-1 Improved glycemic control and HbA_1c_ Improved weight gain Improved acanthosis nigricans	Ovarian juvenile granulosa cell tumor	2.9
De Kerdanet et al, 2015 (13)	1/DS/F	Biallelic	0.5	10.5	8 (IGF-1/IGFBP-3) 0.06	BD/TDS	Reduced mean glucose Decreased insulin and HbA_1c_ No improvement in ALS Increased IGF-1 and IGFBP-3 Improved feeding and weight/height gain Normal neurologic development Increase in subcutaneous fat thickness	Frequent hypoglycemia Transient elevation of liver enzymes Mild nephromegaly with microalbuminuria	>11
Carmody et al, 2016 (19)	1/RMS/F	Biallelic	3.5	5	1.73 units/kg/d	BD	Decreased insulin, HOMA and HbA_1c_ Increased IGF-1 and IGFBP-1 Increased adiponectin Reduced albumin/creatinine ratio No changes in IGFBP-3 Improvement of acanthosis nigricans, hyperkeratosis, and hypertrichosis Reduce tendency to develop ketosis during viral infections	None	>8.5
Kostopoulou et al, 2016 (20)	1/DS/F	Biallelic	0.25	NA	0.9	NA	Improved glucose control	None	8.33
Plamper et al, 2018 (21)	1/DS/M	Biallelic	0.67	14	0.5	BD/CSI	Improved glucose control Decreased HbA_1c_ Weight gain Moderate improvement in motor function and muscular strength	Adenoid hypertrophy with intermittent nocturnal oxygen requirement	1.83
Ferrito et al, 2020 (22)	1/DS/F	Biallelic	3.7	28	0.16	BD	Reduced insulin No improvement in HbA_1c_ Improved growth, muscular tropisms, hypertrichosis, acanthosis nigricans and clitoromegaly	Some concerns about tooth enlargement and adenoid hypertrophy	>6

Abbreviations: ALS, acid labile subunit; BD, 2 daily doses; CSI, continuous subcutaneous infusion; DS, Donohue syndrome; F, female; HbA_1c_, glycated hemoglobin A_1c_; HOMA, homeostatic model assessment; IGF-1, insulin-like growth factor-1; IGFBP, insulin-like growth factor binding protein; M, male; NA, not available; OD, once daily; QDS, 4 daily doses; rhIGF-1, recombinant human insulin-like growth factor-1; RMS, Rabson-Mendenhall syndrome; TDS, 3 daily doses.

^*a*^The diagnostic label used in the original report. According to the survival time, all individuals with a survival greater than 5 years could be more appropriately defined as having RMS form.

### Patient 1

The early clinical course of this patient has been reported previously (12). He was born at 36 weeks’ gestation to consanguineous parents with a birth weight of 1.1 kg (−4.67 SDS). Three siblings with severe IR had died at ages 6.5, 1, and 2 years, respectively. He was shown to have severe hyperinsulinemic hyperglycemia and typical features of insulin receptoropathy.

He received rhIGF-1 (0.1 mg/kg/day) treatment from age 22 months, with improvement in fasting glucose, insulin, HbA1c, and growth and motor development. Genetic analysis confirmed the homozygous p.Arg1119Gln (p.Arg1092Gln) missense variant in the tyrosine kinase domain of the *INSR*, which had been previously reported (12). rhIGF-1 treatment was stopped at age 5 years by parental report due to lack of availability. Between ages 9 and 13 years, the patient experienced recurrent chest infections and severe ketoacidosis. He was transferred to our care at age 13.5 years having developed a further episode of severe ketoacidosis. Intravenous (IV) insulin (up to 10 units/kg/hour) had no effect on blood glucose (BG) concentrations. However, IV fluids containing up to 40 mmol/kg/day of sodium bicarbonate counteracted the metabolic acidosis, allowing progressive weaning of the fluids over 3 weeks, with further stabilization of the acid-base status on oral sodium bicarbonate (11 mmol/kg/day). His weight was 12.4 kg (−3.48 SDS) with a height of 99.8 cm (−7.11 SDS). On examination, he had marked hypertrichosis, extensive acanthosis nigricans, large poorly calcified canine teeth, bilateral cataracts, and a distended abdomen with firm hepatomegaly. He had Tanner stage 2 genitalia and pubic hair with 2-mL testes bilaterally. Heart and renal ultrasound showed mild biventricular hypertrophy with pulmonary hypertension and nephrocalcinosis, respectively. He had a tracheostomy that had been inserted to alleviate subglottic stenosis resulting from repeated intubation. Fasting investigations performed after recovery from acute metabolic decompensation confirmed hyperinsulinemic hyperglycemia (BG 23.6 mmol/L; insulin 502.8 pmol/L) with inappropriately elevated nonesterified fatty acids (NEFAs) and β-hydroxybutyrate (β-OHB) concentrations (1.04 and 3.63 mmol/L, respectively), consistent with adipose and hepatic IR, which was no longer adequately compensated for by hyperinsulinemia. Serum IGF-1 and insulin-like growth factor binding protein-3 (IGFBP-3) concentrations were undetectable. Fasting IGFBP-1 was elevated (255 ng/mL; normal adult range, 7-38 ng/mL), while IGF-2 concentration was slightly reduced (528 ng/mL; normal adult range, 553-898 ng/mL). Fasting triglycerides were moderately elevated, with a normal total cholesterol and a low high-density lipoprotein cholesterol (Table 3). HbA_1c_ was 181 mmol/mol.

**Table 3:** Fasting biochemical profile of the 4 patients

	Patient 1	Patient 2	Patient 3	Patient 4	Normal range
**Age, y**	14.3	17.24	0.5	0.8	NA
**Liver function tests**					
ALP, U/L	**1069**	**2730**	**487**	234	80-425
ALT, U/L	**38**	**40**	**187**	**42**	0-32
Albumin, g/L	**24**	**31**	**32**	**23**	34-42
Total bilirubin, μmol/L	**180**	17	**71**	**65**	<18
Conjugated bilirubin, μmol/L	**146**	NA	**27**	2	<10
**Lipids**					
Cholesteroll, mmol/L	4.50	**8.4**	NA	3.80	1.0-5.0
Triglycerides, mmol/L	**2.94**	0.79	NA	1.30	0.5-2.0
HDL cholesterol, mmol/L	0.50	NA	NA	NA	
LDL cholesterol, mmol/L	2.66	NA	NA	NA	
VLDL cholesterol, mmol/L	1.34	NA	NA	NA	
**Urea, electrolytes**					
Urea, mmol/L	1.1	1.4	1.4	2.5	0.7-5
Creatinine, μmol/L	**48**	27	**12**	15	13-32
Sodium, mmol/L	143	140	136	137	133-146
Potassium, mmol/L	3.9	3.6	4.2	3.4	3.2-6
Corrected calcium, mmol/L	**2.80**	2.30	2.37	2.33	2.17-2.44
**Renal tubular screen**					
Albumin/creatinine	**82.5**	**144.0**	**274.0**	**96.3**	1.7-12.2
RBP/creatinine	**13 647**	**44 417**	289	NA	1.5-448
NAG/creatinine	**549**	**1088**	**1510**	NA	2-27
Calcium/creatinine	**5.07**	**5.60**	0.60	2.11	0.09-2.2
Magnesium/creatinine	**2.50**	**5.03**	0.42	**<0.00**	0.4-2.2
Phosphate/creatinine	**21.50**	**32.12**	**19.96**	**<0.02**	1.2-19
TRP, %	**52**	**39**	**68**	88	70-100
**Coagulation, post vitamin K**					
PT, s	10.0	11.4	**16.1**	**17.0**	9.6-11.8
aPTT, s	**39.5**	34.9	**46.5**	**57.8**	26-38
Fibrinogen, mg/dL	3.7	2.0	1.6	**0.8**	1.7-4.0
Factor II, IU/dL	NA	93	56	**25**	50-150
Factor V, IU/dL	NA	**187**	59	97	50-150
Factor VII, IU/dL	NA	77	**26**	**14**	50-150
Factor VIII, IU/dL	**171**	113	125	133	50-150
Factor IX, IU/dL	88	**41**	**20**	**13**	50-150
Factor X, IU/dL	NA	122	75	51	50-150
Factor XI, IU/dL	91	79	**37**	**23**	50-150
Factor XII, IU/dL	70	92	71	**34**	50-150

Values above or below the reference ranges are highlighted in bold.

Abbreviations: ALP, alkaline phosphatase; ALT, alanine transaminase; aPTT, active thromboplastin time; HDL, high-density lipoprotein; LDL, low-density lipoprotein; NA, not available/not applicable; NAG, *N*-acetylglucosamine; PT, prothrombin time; RBP, retinoid binding protein; TRP, tubular reabsorption of phosphates; VLDL, very low-density lipoprotein.

Further investigations revealed normal adrenal and thyroid function, low testosterone (0.7 nmol/L), prepubertal response to gonadotropin-releasing hormone stimulation (peak luteinizing hormone <0.7 IU/L, peak follicle-stimulating hormone 1.5 IU/L), and normal sex hormone–binding globulin 86 nmol/L (normal range [NR], 40-137 nmol/L). An IGF-1 generation test (33 μg/kg/day rhGH administered over 4 consecutive days) showed no increase in IGF-1 or IGFBP-3 concentrations. His bone age was 7.4 years (chronological age, 13.5 years).

At age 14.5 years, he was commenced on rhIGF-1 at 0.04 mg/kg/day once daily. A biochemical profile revealed modest reductions in glucose, insulin, and IGFBP-1 concentrations during the 6 hours after rhIGF-1 administration. The dose of rhIGF-1 was progressively increased to 0.64 mg/kg/day. Two months later there was a reduction in the glucose, HbA_1c_ (102 mmol/mol), NEFA and β-OHB concentrations, with a weight increase of 1.3 kg. Four months later, however, he died in his sleep. No autopsy was performed per his parents' wishes and thus no cause of death was identified. As rhIGF-1 was administered in the morning, acute rhIGF-1-associated hypoglycemia is unlikely to have been the cause of death.

### Patient 2

This male infant was born at term to nonconsanguineous parents, with a birth weight of 2.18 kg (−1.41 SDS), a length of 45 cm (−1.27 SDS), and a cranial circumference of 34.5 cm (−1.7 SDS). He had intrauterine growth restriction (IUGR) from 33 weeks of gestation. Soon after birth he was noted to have fasting hypoglycemia (BG 2.0 mmol/L) and postprandial hyperglycemia (BG up to 15 mmol/L), with a fasting insulin of 6570 pmol/L. He experienced transient respiratory distress, cholestatic jaundice, and failure to thrive. He had marked acanthosis nigricans, paucity of adipose tissue, breast tissue hyperplasia, large low-set ears, thick lips, and large hands and genitalia. Abdominal and heart ultrasound showed bilateral nephromegaly with nephrocalcinosis and septal hypertrophy, respectively. Genetic analysis of the *INSR* gene revealed compound heterozygosity for a paternally inherited, previously studied missense variant in the extracellular L1 domain of the receptor p.Ile146Met (p.Ile119Met) (9), and a maternally inherited nonsense variant in the intracellular tyrosine kinase domain, p.Arg1066X (p.Arg1039X).

At age 4 months, his tolerance to fasting was 1 to 2 hours, mandating continuous feeding via gastrostomy. Fasting investigations revealed normal liver and kidney function (see Table 3), HbA_1c_ 30 mmol/mol, undetectable IGF-1 and IGFBP-3, and elevated IGFBP-1 (212 ng/μL; NR, 7-38 ng/μL). Urinary calcium/creatinine ratio was 3.5 (0.09-2.2). Hypoglycemia screening (Table 4) revealed a high insulin, with very high molar ratio of insulin:C peptide, characteristic of insulin receptor defects, along with suppressed NEFA and β-OHB concentrations. An IGF-1 generation test showed absent IGF-1, IGFBP-3, and acid labile subunit responses to GH.

**Table 4:** Hypoglycemia screening at diagnosis in patient 2 and patient 4

	Patient 2	Patient 4
**Age, y**	0.5	0.2
Blood glucose, mmol/L	1.7	2.3
Insulin, pmol/L	5069	>2083
C-peptide, pmol/L	2995	—
NEFAs, mmol/L	0.64	0.3
β-Hydroxybutyrate, mmol/L	<0.05	0.05
Cortisol, nmol/L	834	588
GH, mcg/L	1.33	0.7
Ammonia, μmol/L	75	60
Lactate, mmol/L	2.3	3.1
Pyruvate, mmol/L	0.09	0.039
Acylcarnitine	Normal	Normal
Plasma amino acids	Normal	Normal
Glucagon, pmol/L	32	—
Proinsulin, pmol/L	51	—
Urine organic acids	Normal	Normal
Urine ketones	Absent	Absent

Abbreviations: GH, growth hormone; NEFAs, nonesterified fatty acids.

**Figure 1:**
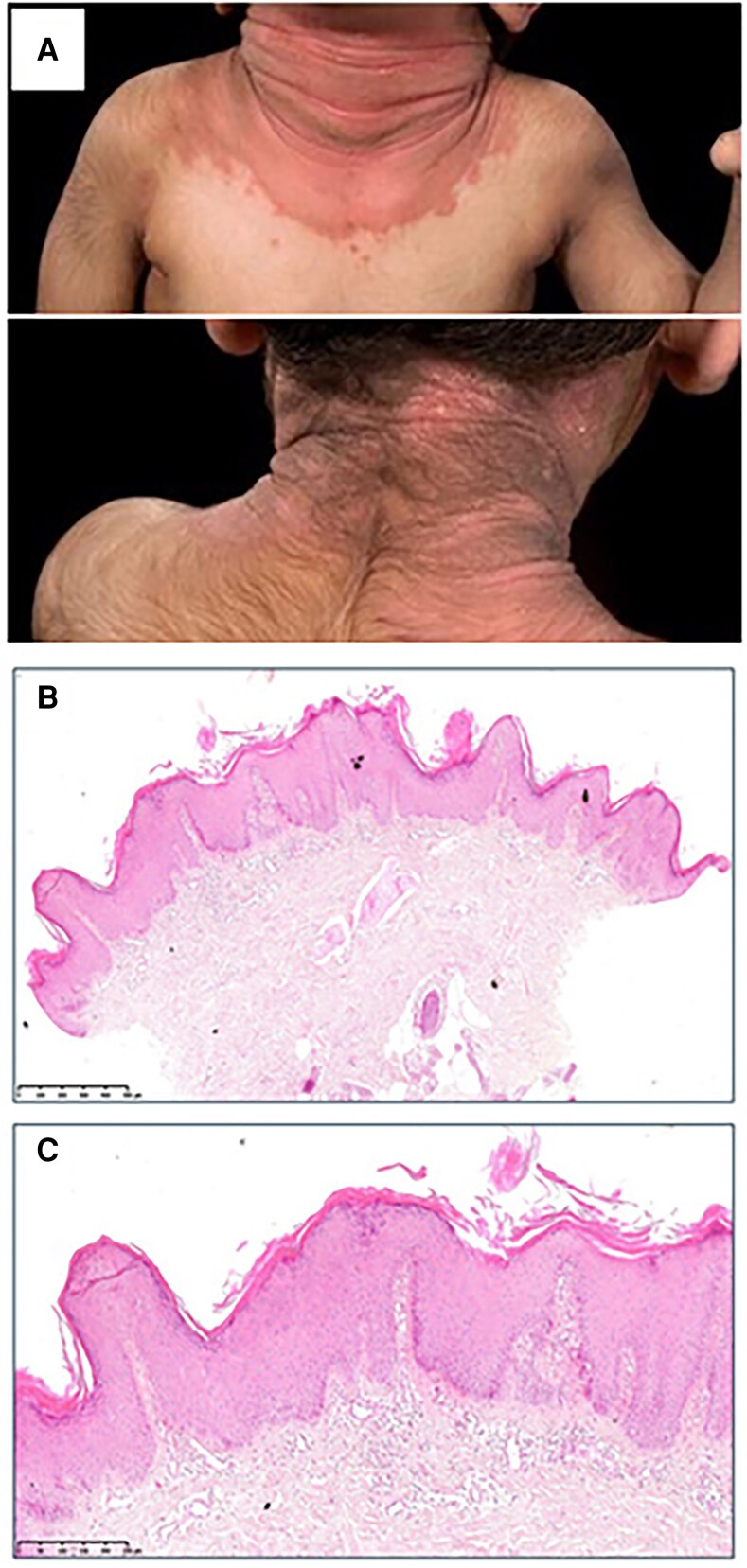
Dermatological features in patient 2. A, Appearance of skin at about 2 years; B and C, Hematoxylin and eosin–stained skin biopsy appearance at age 7 years. The epidermis is papillomatous and verrucous, with a compact orthokeratotic horny layer, occasionally showing parakeratosis. The dermis is richly vascularized and contains a moderate inflammatory infiltrate of lymphocytes and histiocytes. The overall features are suggestive of a keratinization disorder. Scale bars are B, 500 mm and C, 250 mm.

At age 9 months, the patient was commenced on rhIGF-1/IGFBP-3 (SomatoKine), at a starting dose of 0.1 mg/kg/day once daily. Four months later he developed diffuse, pruritic, ichthyosiform erythroderma, more severe in his neck, chest, and around the nose and ears, and complicated by recurrent skin infections (Fig. 1A). The skin lesions did not respond to topical tacrolimus and emollients, but slightly improved with topical acitretin and courses of antibiotic and oral antihistamine.

**Figure 2:**
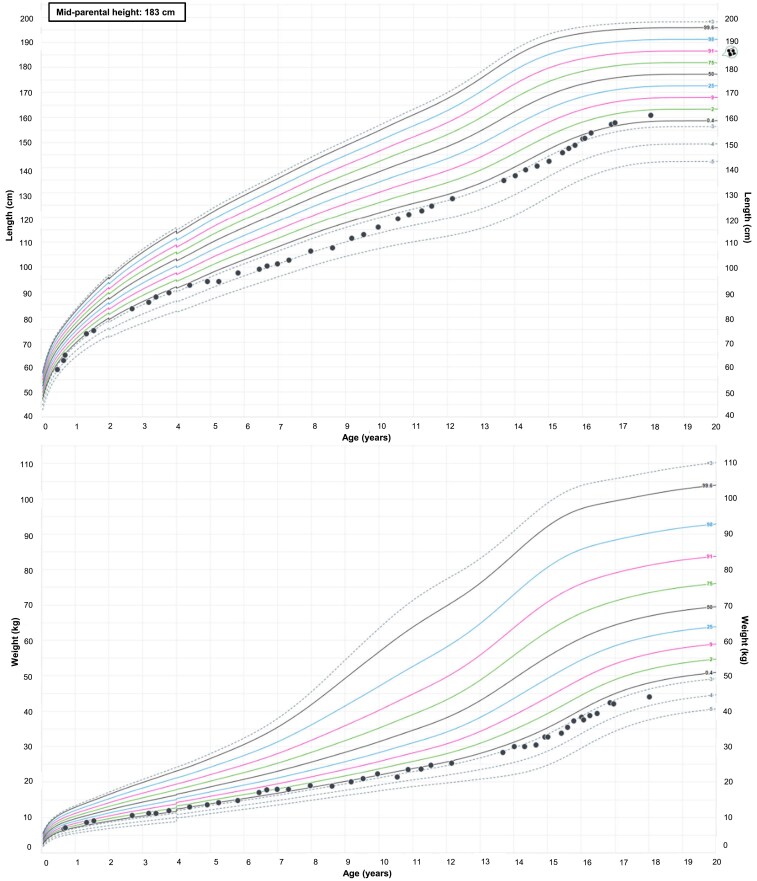
Growth chart of patient 2, showing a dose-dependent growth-promoting effect of recombinant human insulin-like growth factor-1, even if the growth velocity remained suboptimal and the near-final height lies below the mid-parental height.

**Figure 3:**
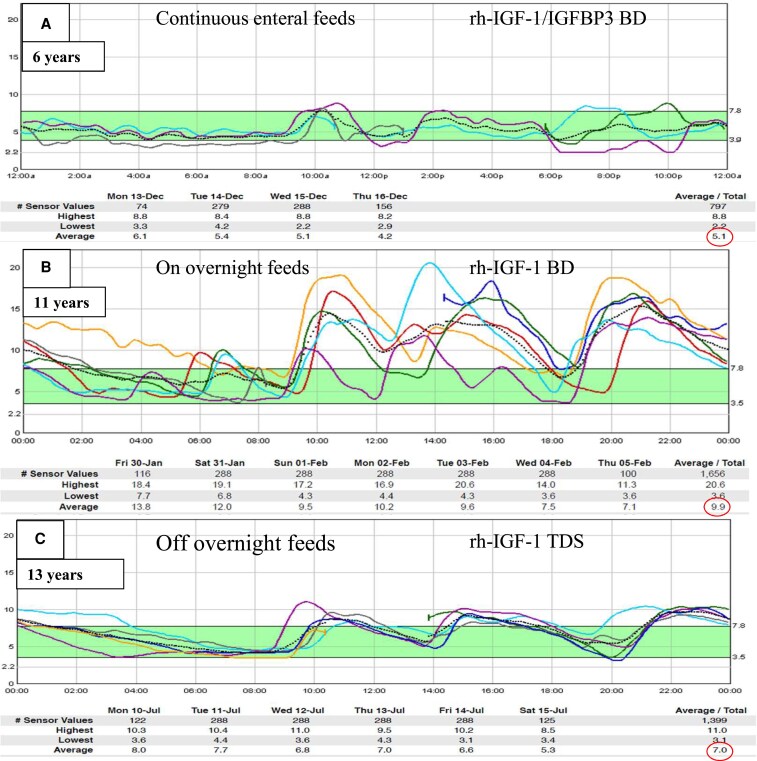
Changes in blood glucose profile over time in patient 2, A, highlighting poor fasting tolerance and tendency to develop hypoglycemia at age 6 years, with B, subsequent switch to diabetes mellitus. The graph demonstrates the clear insulin-like effects of recombinant human insulin-like growth factor-1 (rhIGF-1) therapy, specifically showing how the clinical adjustment of the rhIGF-1 regimen from B, 2 daily doses to C, 3 daily doses effectively allowed for a sustained reduction in the average blood glucose concentration, as indicated by the circled area.

After initial catch-up, his growth slowed by age 4 years (Fig. 2), despite a gradual increase in the dose of SomatoKine. He also suffered from glue ear. At age 4 years, his fasting tolerance had improved to 4 hours. Hypoglycemia screening revealed detectable NEFAs and β-OHB in spite of raised insulin, indicating early decompensation of endogenous mechanisms preventing ketoacidosis. Evolution of his glycemic profile, treatment, and feeding regimens is shown in Fig. 3. At age 8 years, he was switched to rhIGF-1 (0.15 mg/kg/day in 2 divided doses) because SomatoKine was no longer available. Following injection of rhIGF-1, he achieved IGF-1 concentrations of 500 ng/mL and 100 ng/mL at 2 and 8 hours post dose, respectively. His fasting tolerance further increased to approximately 6 hours at age 10 years, permitting switching of his feeding regimen to overnight continuous feeds. The dose of rhIGF-1 was gradually increased to 0.3 mg/kg/day in 3 divided doses. At age 12 years, he was able to fast for 12 hours, and overnight feeds were discontinued. Two years later, his mean postprandial BG concentrations were greater than 20 mmol/L (HbA_1c_ 55 mmol/mol), so metformin was commenced and gradually increased to 1.5 g daily. He entered puberty at age 12.3 years.

At age 7 years, while still taking SomatoKine, persisting erythrodermic skin lesions led to reevaluation. A skin biopsy revealed verrucous epidermal hyperplasia with orthokeratotic or occasionally parakeratotic hyperkeratosis, with focal microabscesses (see Fig. 1B and 1C). Staining for lympho-epithelial Kazal-type-related inhibitor (LEKTI) and transglutaminase was normal. Appearances were adjudged nonspecific, although some features overlapped with those of conditions such as inflammatory linear verrucous epidermal nevus, and psoriasis. Treatment with urea-containing emollient and amoxicillin led to improvement of skin manifestations.

At age 18 years, the patient’s height was 160.8 cm (−2.33 SDS), with a growth velocity of 2.2 cm/year (see Fig. 2), Tanner stage 4 genitalia, and pubic hair with 20- to 25-mL testes bilaterally. His skin had improved considerably. Due to daytime hyperglycemia, and an increase in HbA_1c_ to 85 mmol/mol, he was commenced on U500 insulin. Over the following 2 years, mecasermin was weaned as insulin dose increased, and finally stopped at age 20.2 years. Echocardiography on cessation of mecasermin remained normal. Around this age skin problems flared again, but improved with acitretin (1 mg/kg). At the time of writing, therapy with biologic agents is being discussed in the event of recurrence or new flares resistant to acitretin.

At age 20.2 years, with weight of 49.5 kg (−2.88 SDS), height of 164 cm (−1.61 SDS), body mass index of 18.4 (−1.78 SDS), and HbA_1c_ of 75 mmol/mol on 780 units U500 insulin per day, subcutaneous metreleptin was started at 2.5 mg once daily. It was stopped after 4 months in the face of 4 kg weight loss, albeit with mildly improved glycemia (HbA_1c_ 72 mmol/mol). On stopping metreleptin, the patient’s weight rapidly increased by 3 kg in 3 to 4 weeks. At the time of writing, at age 20.8 years, insulin dose titration continues, now at 1200 units per day (25.5 units/kg/day), with a predicted HbA_1c_ of 57 mmol/mol.

### Patient 3

This male infant was born to consanguineous parents at 38 weeks gestation by cesarean delivery because of IUGR, weighing 1.2 kg (−5.68 SDS). Endocrine evaluation was sought at age 5 months because of growth failure and features characteristic of DS (triangular face, thick lips, small nose, prominent ears, distended abdomen, large phallus, rectal prolapse, left sided inguinal hernia, hirsutism). On admission, his BG ranged between 2.0 and 11.5 mmol/L, with fasting insulin concentrations greater than 2080 pmol/L, requiring continuous enteral nutrition.

His liver function was disturbed (see Table 3), with persistently elevated alanine transaminase and prolonged coagulation times that did not improve after IV vitamin K. An extensive diagnostic work-up failed to identify a cause of liver dysfunction. An ultrasound of the abdomen showed enlarged kidneys with nephrocalcinosis and marked ascites, with initially normal liver echotexture. He developed small focal liver lesions around age 1 year, which were confirmed on magnetic resonance imaging (MRI). He exhibited an albumin/creatinine ratio higher than expected based on low creatinine concentrations, and strong nonspecific loss of amino acids consistent with proximal renal tubular dysfunction, without significant electrolyte loss (see Table 3). Echocardiogram revealed biventricular hypertrophy with left ventricular outlet obstruction.

Endocrine work-up showed a high sex hormone–binding globulin of 315 nmol/L (15-48), undetectable IGF-1 and IGFBP-3, and low-normal free thyroxine (FT4) concentration (10.1 pmol/L; NR, 10.2-20.6 pmol/L), with an inappropriately low thyrotropin (TSH) (1.5 mU/L), that required levothyroxine treatment. MRI of the brain was normal.

*INSR* sequencing demonstrated a homozygous missense variant in the extracellular L1 domain, namely p.Gly111Glu (p.Gly84Glu). An IGF-1 generation test showed no significant increase in serum IGF-1 and IGFBP-3 and, at age 6 months, he was commenced on 0.1 mg/kg/day rhIGF-1 in 2 divided doses. Following injection of rhIGF-1, IGF-1 concentrations peaked at 52 ng/mL at 2 hours, but were undetectable after 8 hours; insulin concentrations reached a nadir of 1347 pmol/L. However, after 3 months, the treatment was stopped due to worsening of comorbidities and frequent hypoglycemia. The infant experienced multiple episodes of sepsis and developed a duodenal ulcer. He had a cardiac arrest during a central line insertion and died at age 10.5 months of acute respiratory failure.

### Patient 4

This male infant was born at 35 + 2 weeks to consanguineous parents by cesarean delivery for severe IUGR from 20 weeks of gestation with fetal bradycardia. His birth weight was 1.094 kg (−4 SDS) with a length of 44 cm (−1.25 SDS).

He had persistent hyperglycemia despite low glucose delivery and administration of insulin, and typical dysmorphic features (triangular face, thick lips, prominent ears, distended abdomen, large phallus, thick skin). His fasting insulin concentrations were greater than 13 800 pmol/L, with C-peptide greater than 7000 pmol/L and an undetectable IGF-1. He also suffered from 2 episodes of sepsis and a cardiac arrest during anesthesia.

He was transferred to our care at age 1.5 months. His fasting tolerance was 3 hours with BG concentrations ranging from 1.7 to 23.3 mmol/L, requiring continuous enteral feeds. Biochemical evaluation revealed a hemoglobin of 8 mg/dL with a low reticulocyte count, plasma sodium of 125 to 131 mmol/L, and potassium of 2.5 mmol/L, requiring oral electrolyte supplements. Serum albumin concentration was low, with no increased urinary or gastrointestinal loss (see Table 3). His coagulation was disturbed (see Table 3). Coagulation times normalized with a 50:50 mixing test, but coagulation factor concentrations did not improve after intravenous vitamin K, suggesting synthetic dysfunction.

Endocrine testing showed secondary hypothyroidism requiring levothyroxine therapy (FT4 8.8 pmol/L; NR, 10.2-20.6 pmol/L), TSH 0.7 mU/L), undetectable IGF-1 and IGFBP-3, raised IGFBP-1 at 198 μg/L (NR, 0.6-14.4 μg/L), normal IGF-2 of 8.2 nmol/L (NR, 2-36 nmol/L), HbA_1c_ of 41 mmol/mol, and a urine calcium/creatinine ratio of 5.15. MRI showed normal brain and pituitary morphology. Hypoglycemia screening (see Table 4) revealed insulin greater than 20 800 pmol/L, GH of 0.7 mcg/L, lactate of 2.1 mmol/L, acetoacetate less than 0.03 mmol/L, NEFAs of 0.3 mmol/L, β-OHB of 0.05 mmol/L, cortisol of 588 nmol/L, and undetectable IGF-1 and IGFBP-3. An IGF-1 generation test demonstrated no increase in IGF-1.

Echocardiography showed mild pulmonary valve stenosis, a small patent foramen ovale, and mild left ventricular hypertrophy. Abdominal ultrasound showed multiple small liver hemangiomas and enlarged kidneys with nephrocalcinosis.

Genetic analysis of the *INSR* gene revealed a homozygous p.Trp642Arg (p.Trp615Arg) variant in the extracellular FnIII-2A domain. At age 3 months, he was commenced on 0.08 mg/kg/day rhIGF-1 in 2 divided doses. Within 3 months of the start of therapy, his weight improved, BG stabilized with bolus enteral feeds, and HbA_1c_ decreased to 33 mmol/mol.

At age 18 months, the patient experienced fungal endocarditis, causing multiple cerebral infarctions. By age 2 years, despite receiving 0.27 mg/kg/day rhIGF-1 in 3 divided doses, his BG concentrations ranged between 7 and 15 mmol/L (HbA_1c_, 58 mmol/mol). He died at age 2.3 years from wound dehiscence and airway complications after abdominal surgery for a prolapsed gastrostomy.

## Discussion

The results of our study expand the phenotypic spectrum of DS and RMS and highlight unreported challenges in their management. Given the rarity and poor life expectancy of these syndromes, availability of data on long-term survivors is essential to guide the management of other cases. Autosomal recessive forms of IR show a continuum of severity, in which DS and RMS are at the most extreme end. DS is usually associated with death during the first years of life, whereas RMS is associated with longer survival, but the demarcation is not precise, and older labels such as leprechaunism are common in the historical literature (1). In both DS and RMS, insulin receptor function is severely impaired, likely to below 10% in all cases. The precise degree of residual receptor function is believed to determine longevity, but no sufficiently detailed functional studies of reported receptor variants have been undertaken to establish this robustly. In the present study we defined patient 2 as having an RMS phenotype based on his clinical course, and the demonstrable residual activity of one of his compound heterozygous *INSR* mutations (1, 9). Treatment options for syndromes of extreme IR are limited, and no controlled trials have been undertaken due to the rarity of the condition. High-dose insulin is the first-line treatment for ketoacidosis. Although metreleptin and metformin can improve glycemic control in patients with RMS phenotype, these do not allow achievement of a fully satisfactory metabolic control (10-12), and have not been studied in patients with a DS phenotype. Despite variable results (5-8, 13-22) (see Table 2), rhIGF-1 is the only therapeutic agent reported to elicit reproducible metabolic benefits, especially when given at high doses or as a continuous subcutaneous infusion (5, 15, 23). On the other hand, reporting bias may favor positive outcomes. The rationale behind its use in these syndromes is the ability of IGF-1 to bind to the IGFR, INSR, and INSR/IGFR hybrid receptors on target cells (5, 6), influencing the post–receptor crosstalk between insulin and IGF-1 signaling pathways (1).

To date only 12 patients with extreme IR receiving rhIGF-1 for at least 4 months have been reported in the literature (4-7, 10, 13-15, 17-22) (see Table 2). Among these, 10 carried biallelic mutations of *INSR* (4-6, 10, 13-15, 17-22), while the other 2 had indeterminate genotypes and some overlapping features of severe insulin receptoropathy (7). In one case (4), no substantial treatment outcomes have been reported. The long-term outcomes of rhIGF-1 treatment are not consistent between these studies. Indeed, some but not all studies reported improvements in glycemic control, linear growth, weight, and neuromotor function (see Table 2). The results of a recent systematic review (8) assessing the outcomes of rhIGF-1 or IGF-1/IGFBP-3 combined treatment beyond 28 days in patients with *INSR* pathogenic variants found an improvement of HbA_1c_ only, which was more marked in monoallelic (n = 6), compared to biallelic forms (n = 7) (least square mean reduction 2% vs 1.5%, respectively). On the other hand, hypoglycemia episodes and one case of soft tissue overgrowth were reported.

We report outcome data of 3 further male patients with biallelic forms, in addition to providing the long-term clinical course of a patient already described (6). By doing so we provide further insights regarding the real benefits and the risks of rhIGF-1 in the long term. As shown in our cases, although rhIGF-1 cannot prevent the progressive decompensation to insulin-resistant diabetes mellitus, its insulin-like effects allow modest improvement of glycemic control, especially when given more frequently, up to 3 times daily (see Fig. 3). However, improved glucose metabolism cannot be certainly attributable to rhIGF-1 alone, as other concomitant metabolic changes occurring at various ages (including puberty initiation) might play a role.

DS and RMS exhibit variability in the expression and severity of comorbidities associated with IR. Growth failure is a constant feature of severe IR. In vitro growth of fibroblasts from patients with genetic forms of IR seem to be impaired in a manner proportional to the severity of the phenotype (18). As confirmed in our patients, growth restriction commences in utero. Data both from human and murine models suggest that, in addition to absent mitogenic actions of insulin, IUGR may be caused by a lack of IGF-1/IGFBP-3 (5, 24, 25) and/or by reduced effects of IGF-2 via the INSR (26). Indeed, in our patients, IUGR was noted by the end of the second trimester, when IGF-2 seems to have more relevant actions on growth (24).

The pathophysiology of GH insensitivity associated with insulin-resistant syndromes is likely multifactorial. Insulin exerts permissive actions on GH receptor signaling, enhancing the transcription of the *IGF1* gene (27-31). While these actions are partly exerted at the receptor level (32), in vitro studies using hepatocytes (33) and fibroblasts from severely insulin-resistant patients (15) also suggest a post–receptor defect in GH signaling. Moreover, as suggested by our and other reported cases (5, 19, 34-36), reduced IGF-1 bioavailability may result from continued perturbation of IGFBP-3 and IGFBP-1 concentrations postnatally. As insulin promotes IGFBP-3 gene expression (37), loss of insulin receptor function may reduce IGFBP-3 biosynthesis, accelerating IGF-1 urinary excretion. IGFBP-1 is also highly insulin responsive (38-40) and in vitro, in vivo, and transgenic murine (41) studies have shown that high IGFBP-1 concentrations may result in growth impairment due to impaired receptor binding by IGF-1 (42) or impaired response to GH or IGF-1 (43). We observed a decrease in IGFBP-1 (patient 1) after administration of rhIGF-1, most likely acting via the IGFR. Interestingly, as previously reported (44), in addition to GH insensitivity, patients 2 to 4 also exhibited inappropriately low GH responses to hypoglycemia, suggesting dysregulated GH production. Postnatally, endocrine mechanisms of linear growth impairment may be exacerbated by urinary and/or intestinal losses, recurrent infections, and organ failure (45).

By providing data on long-term outcomes, the results of our study substantially contribute to clarify that rhIGF-1 treatment exerts some growth-promoting effects in severely insulin-resistant patients with no severe comorbidities; however, it is unable to normalize growth so that the final height of these patients invariably lies below normal (see Table 1 and Fig. 2). One possible explanation for this is the inhibitory action of excess insulin or defective INSR on IGF-1 receptor signaling (18). Dose optimization also plays a key role, with the most impressive results being reported with longer-term use of very high doses (4-7, 10, 13-15, 17-22) (see Table 2). In this respect, our cases indicate the need for testing IGF-1 half-life in vivo periodically, thus allowing tailored treatment aiming at maintaining plasma IGF-1 concentrations as far as possible within the NR.

DS patients are resistant to ketoacidosis during the first years of life, possibly due to loss of GH-mediated lipolysis and ketogenesis due to GH deficiency (46, 47) or insensitivity (48) and/or action of excess insulin on IGFR persistently expressed in immature liver (46). The latter may also contribute to fasting nonketotic hypoglycemia in DS (1), which may, at least in part, account for the developmental delay associated with DS (49). Progression to severely insulin-resistant diabetes mellitus with attendant emergence of ketoacidosis beyond the early years of life is still unexplained but likely reflects secondary failure of β-cell hypersecretion of insulin (1) and/or progressive disappearance of IGFR from the liver. Further, not mutually exclusive, possibilities are improved GH sensitivity as insulin concentrations fall, or selective resistance to IGF-1 generation, with intact lipolytic and ketogenic responses to GH.

Although hypothyroidism has been already described in DS (4, 44, 50), its pathogenesis remains uncertain. Despite FT4 concentrations being positively related to insulin sensitivity (51), data from our and other reports documented TSH concentrations inappropriately low for the degree of hypothyroxinemia (23, 44) or even undetectable (4), indicating secondary hypothyroidism. Co-occurrence of secondary hypothyroidism, dysregulated GH production, and delayed puberty may suggest a negative effect of severe IR on hypothalamo-pituitary crosstalk. Indeed, in one report (23) rh-IGF-1 therapy alone resulted in improved hypothyroxinemia because of improved metabolic condition. One of the factors possibly explaining hypothalamo-pituitary dysregulation (especially delayed puberty) in the context of severe receptoropathies is leptin deficiency, due to the scarcity of adipose tissue (52).

Patient 2 developed a chronic diffuse psoriasiform skin rash several months after starting rhIGF-1/IGFBP-3 treatment. A similar, but apparently less extensive, rash developing 3 months after commencement of rhIGF-1 treatment was previously reported by Grasso et al (4) in an infant with biallelic missense *INSR* mutations resulting in an RMS phenotype associated with Bartter-like syndrome. The delayed onset of the rash on starting rhIGF-1, and its exacerbation later following withdrawal of rhIGF-1, renders it unlikely to have been a side effect of rhIGF-1 treatment. However, previous findings in the psoriatic epidermis of increased IGF-1 receptor expression in proliferative areas (53) and elevated IGFBP-3 concentration in the basal layer (54) may suggest a potential link between skin rashes and IGF-1 or IGFBP-3 administration.

Early-onset bilateral cataracts, as well as the increased risk of sepsis associated with extreme IR, are both likely consequences of chronic hyperglycemia and immune dysfunction (55-57).

Numerous reports attest that liver involvement is common in DS, but its spectrum is poorly defined, ranging from mild manifestations (ie, transient cholestatic jaundice) to liver failure. Hepatic GH insensitivity and/or a perturbation of growth factors may all contribute to the associated liver disease (10, 58, 59). In this context, we provide evidence that deficiencies in liver synthesis of key physiological factors (eg, albumin, coagulation factors) also have functional effects (patients 3 and 4).

In conclusion, we provide long-term clinical data regarding syndromes of extreme IR, expanding the phenotypic spectrum of DS and RMS. Controlled studies for such rare conditions cannot be undertaken, and thus data from clinical experience are crucial to guide management. In the present study more evidence has been gathered in an unbiased, prospective manner, clarifying that the benefits of rhIGF-1 treatment in terms of metabolic control and linear growth remain largely unsatisfying. This work highlights the need for more definitive therapeutic options for biallelic insulin receptoropathy. Genome editing or gene replacement seem an appealing option at face value, given rapid advances for other rare genetic diseases featuring organ-autonomous functional defects, including, for example, hepatic metabolic disorders and β-cell failure (60, 61). However insulin receptoropathy is a systemic disorder in which metabolic crosstalk among tissues is severely perturbed. If a defective *INSR* gene were replaced or corrected in the liver, then the cells with the intact receptor would be exposed to extremely high levels of insulin driven by dysfunction elsewhere, with potential adverse effects on cell metabolism and/or growth. Thus, genetic approaches to receptor defects are likely to require multiorgan or systemic approaches. Another approach that is attracting renewed interest is the development of novel insulin receptor ligands that are able to engage and activate the subset of mutant receptors that are expressed at the cell surface (62, 63). While such a strategy relies on discerning which variants do show cell surface expression, this is being facilitated by approaches such as deep mutational scanning of the *INSR* gene (64). Finally, some small molecule–based strategies have been described allowing post–receptor activating of metabolic signaling (65, 66). There is thus a portfolio of approaches offering therapeutic promise for the future for extreme congenital receptoropathy.

## Data Availability

All datasets on which the conclusions of the paper rely are available to editors and reviewers on request. Data are not publicly available for ethical reasons. Further inquiries can be directed to the corresponding author.

## Abbreviations

β-OHB: β-hydroxybutyrate
BG: blood glucose
CGMS: continuous glucose monitoring system
DS: Donohue syndrome
FT4: free thyroxine
GH: growth hormone
HbA_1c_: glycated hemoglobin A_1c_
IGF-1: insulin-like growth factor-1
IGFBP: insulin-like growth factor binding protein
IGFR: insulin-like growth factor-1 receptor
*INSR*: insulin receptor gene
IR: insulin resistance
IUGR: intrauterine growth restriction
IV: intravenous
MRI: magnetic resonance imaging
NEFAs: nonesterified fatty acids
NR: normal range
rhIGF-1: recombinant human insulin-like growth factor-1
RMS: Rabson-Mendenhall syndrome
TSH: thyrotropin
